# Dual Roles of microRNA-122 in Hepatocellular Carcinoma and Breast Cancer Progression and Metastasis: A Comprehensive Review

**DOI:** 10.3390/cimb46110711

**Published:** 2024-10-25

**Authors:** Essam Al Ageeli

**Affiliations:** Department of Basic Medical Sciences (Medical Genetics), Faculty of Medicine, Jazan University, Jazan 45142, Saudi Arabia; ealageeli@jazanu.edu.sa

**Keywords:** microRNA-122, hepatocellular carcinoma, breast cancer, metastasis, sorafenib, radiotherapy, ADAM10, triple-negative breast cancer

## Abstract

microRNA-122 (miR-122) plays crucial yet contrasting roles in hepatocellular carcinoma (HCC) and breast cancer (BC), two prevalent and aggressive malignancies. This review synthesizes current research on miR-122’s functions in these cancers, focusing on its potential as a diagnostic, prognostic, and therapeutic target. A comprehensive literature search was conducted using PubMed, Web of Science, and Scopus databases. In HCC, miR-122 is downregulated in most cases, suppressing oncogenic pathways and reducing tumor growth and metastasis. Restoring miR-122 levels has shown promising therapeutic potential, increasing sensitivity to treatments like sorafenib. In contrast, in BC, miR-122 plays a pro-metastatic role, especially in triple-negative breast cancer (TNBC) and metastatic lesions. miR-122′s ability to influence key pathways, such as the Wnt/β-catenin and NF-κB pathways in HCC, and its role in enhancing the Warburg effect in BC underline its significance in cancer biology. miR-122, a key factor in breast cancer radioresistance, suppresses tumors in radiosensitive cells. Inhibiting miR-122 could reverse resistance and potentially overcome radiotherapy resistance. Given its context-dependent functions, miR-122 could serve as a potential therapeutic target, where restoring or inhibiting its expression may help in treating HCC and BC, respectively. The dual roles of miR-122 underscore its significance in cancer biology and its potential in precision medicine.

## 1. Introduction

Hepatocellular carcinoma (HCC) is a major cause of cancer-related deaths, ranking as the sixth most commonly diagnosed cancer and the third leading cause of cancer death [1]. Therefore, comprehensively understanding the molecular anomalies leading to HCC is crucial to developing effective therapies. Breast cancer (BC) also poses a serious risk to public health due to its highly aggressive nature. Understanding the processes leading to the development and progression of BC is important for its treatment. Therefore, novel and effective targets are urgently needed to treat BC [2].

More than 90% of RNAs produced in the human genome are non-coding RNAs (ncRNAs) [3]. They govern various biological activities and are associated with various human diseases, including cancer [3]. Long ncRNAs (lncRNAs) use the same enzyme-manufacturing process as mRNAs and function near (cis-acting) or at a distance (trans-acting) from their site of synthesis [4]. MicroRNAs (miRNAs), a short type of ncRNAs, are crucial in regulating gene expression [5]. Mutations in miRNAs, such as miR-122, play critical roles in tumor formation and act as tumor suppressors or oncogenes [6].

Li et al. revealed miR-122′s role in HCC development [7]. miR-122 levels are significantly lower in hepatocarcinoma tissues than in adjacent non-cancerous tissues. In contrast, patients with metastatic breast cancer (BC) exhibit elevated miR-122 levels in their blood, suggesting that miR-122 is produced by cancer cells and may facilitate their spread to distant sites [8]. Delayed detection and inadequate prognostication pose significant challenges to effective cancer diagnosis and treatment. MiRNAs, specifically miR-122, exhibit potential for cancer detection and prognostication [9].

miRNA research has significantly advanced over the last decade. The dysregulation and aberrant expression of miR-122 are associated with many cancer types, including breast cancer, colon cancer, prostate cancer, cervical cancer, thyroid cancer, and renal cell carcinoma. Thus, miR-122 is a potential diagnostic and prognostic biomarker for human cancer in general [10].

A comprehensive PubMed, Web of Science, and Scopus search identified relevant studies from January 2000 to April 2024. The search terms included “microRNA-122”, “miR-122”, “hepatocellular carcinoma”, “HCC”, “breast cancer”, “progression”, and “metastasis”. Manually searching the retrieved article reference lists yielded additional articles.

After evaluating the titles and abstracts, we examined the complete texts of pertinent studies to assess the papers’ eligibility. Studies that met the following criteria were included: (1) peer-reviewed original research articles or comprehensive reviews; (2) studies on miR-122 in hepatocellular carcinoma or breast cancer; and (3) English articles. Conference abstracts, letters, editorials, studies not directly related to miR-122 in HCC or breast cancer, and duplicate publications were excluded.

The data were extracted from the included studies by utilizing a conventional form. We obtained the author(s), year of publication, study design, sample size, experimental techniques, key findings, and conclusions.

miR-122′s roles in HCC and breast cancer progression and metastasis were examined using narrative synthesis, and its effects on each cancer type’s molecular mechanisms, signaling pathways, and cellular processes were categorized.

In this review, we outline miR-122′s roles in the etiology of HCC and BC. This article presents the recent findings on miR-122′s functions as a tumor suppressor in HCC and metastasis promoter in BC, providing a comprehensive overview of the current research landscape on the contrasting roles of miR-122 in different cancer types.

Furthermore, we focused on hepatocellular carcinoma (HCC) and breast cancer (BC) due to the distinct yet critical roles that miR-122 plays in the progression of these two cancers. miR-122 has been extensively studied in HCC, where it functions predominantly as a tumor suppressor; meanwhile, it has been implicated in promoting metastasis in BC. Understanding miR-122′s dual role in these cancers can provide insights into targeted therapeutic approaches that may apply to other solid tumors.

## 2. Biogenesis and Functions of miRNAs

miRNAs are 22 nt long double-stranded hairpin structures that encode transcripts produced from primary (pri)-miRNAs synthesized by Pol II. They form a substrate for a microprocessor protein complex [11]. This microprocessor contains Drosha, an RNase III enzyme; RNA-binding protein DiGeorge critical region 8; and several other supporting factors, such as p68 and p72 [12]. Drosha clips nucleotides on the stem of pri-miRNA, liberating an approximately 60 nt stem-loop, called the precursor-miRNA (pre-miRNA), in the nucleus [13]. A specific transporter, exportin 5, located on the nuclear membrane, exports this newly generated pre-miRNA to the cytoplasm with the assistance of RAN-GTP [14]. Dicer further processes the pre-miRNA in the cytoplasm. Similar to Drosha, Dicer is an RNase III enzyme associated with auxiliary factor RNA-binding proteins (trans-activation-responsive RNA-binding proteins (TRBP/TARBPs)) [15]. Dicer and the TRBP form a double-stranded miRNA duplex, and the Argonaute protein family selects the guide strand. Then, mature miRNA loads onto the RNA-induced silencing complex to regulate mRNA expression [16].

## 3. miR-122 in HCC: Mechanisms and Tumor Progression

Cirrhotic and high-grade dysplastic nodules are critical precursors to hepatocellular carcinoma (HCC) (Table 1). miR-122 plays a significant role in HCC by targeting various genes and signaling pathways, which influence tumorigenesis, cancer progression, and metastasis [17]. Importantly, miR-122 is downregulated in approximately 70% of HCC cases [18].

## 4. Key Genetic Mutations and Dysregulated Signaling Pathways in HCC

The most commonly mutated gene in hepatocarcinogenesis is the telomerase reverse transcriptase (TERT) promoter, found in 60% of cases, followed by mutations in *TP53* and *β-catenin*, which occur in 25–30% of cases, either individually or in combination [19,20]. Additionally, mutations in genes associated with chromatin remodeling, such as AT-rich interactive domain-containing protein 1A (ARID1A; 13%) and ARID2 (7%), are also frequently observed in HCC [21]. Beyond gene mutations, aberrant signaling in pathways such as *Wnt1/β-catenin*, protein kinase B (Akt)/mechanistic target of rapamycin (mTOR), and mitogen-activated protein kinase (MAPK) contributes to cell cycle deregulation in HCC [22].

## 5. miR-122-Mediated Apoptotic Regulation in HCC

### 5.1. miR-122 Targets Cyclin G1 to Inhibit HCC

**Figure 1:**
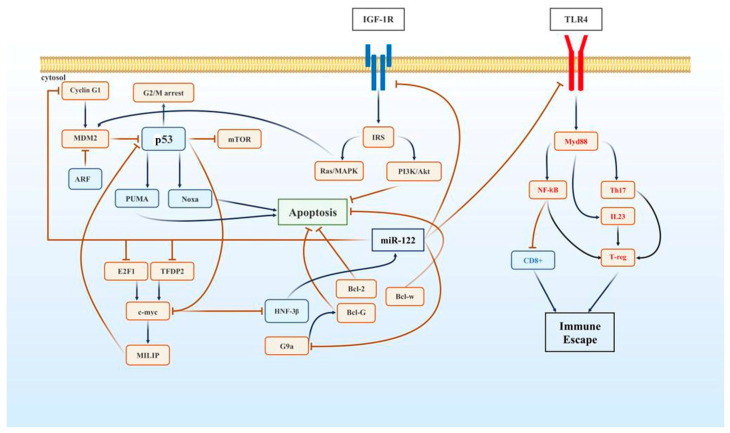
*Cyclin G1*, insulin-like growth factor 1 receptor (IGF-1R), *c-Myc*, *G9a*, and Toll-like receptor 4 (TLR4) are very important for hepatocarcinogenesis as they stop cell death and upregulate the pathways promoting cell growth. *Cyclin G1* and *IGF-1R* activate MDM2, leading to p53 degradation. *c-Myc* upregulates c-Myc-inducible lncRNA inactivating p53 (MILIP) expression and decreases p53 expression. *G9a* activates *Bcl-G*, an antiapoptotic protein. MicroRNA (miR)-122 negatively regulates these mediators by directly interacting with their mRNAs. *TLR4* orchestrates immune escape by activating regulatory T cells (T-regs) and suppressing CD^8+^ activity in tumors.

Resistance to apoptosis is an important mechanism for carcinogenesis, including HCC development. miR-122 targets *cyclin G1*, *Bcl-w*, and *G9a* in HCC, preventing their tumorigenic activity [23]. p53 controls the G2/M cell cycle checkpoint, whereas MDM2 sequesters it for cytoplasmic breakdown. ARF negatively regulates MDM2, temporarily arresting DNA repair [24]. *Cyclin G1*, a non-canonical cyclin, co-precipitates with ARF and MDM2, thus affecting p53. It promotes p53 accumulation for DNA damage repair, but its effect decreases after 72 h [25]. *Cyclin G1* expression is commonly upregulated in HCC; it is a direct target of miR-122 (Figure 1). miR-122 overexpression in HepG2 cells decreases *cyclin G1* levels and radiosensitizes tumor grafts in mice [26].

### 5.2. miR-122 Inhibits HCC by Targeting IGF-1R

*IGF-1R* helps in DNA damage repair and saves damaged cells by stopping apoptosis via MDM2 activation and p53 inhibition in the cytoplasm mediated by *IGF-1* (Figure 1) [27]. The abnormal overactivity of *IGF-1R* promotes stemness in tumor cells and affects the antitumor efficacy of sorafenib in HCC [28]. Additionally, miR-122 directly targets IGF-1R mRNA at the 3′-untranslated region (UTR) and downregulates its expression in HCC cells [23].

### 5.3. miR-122 Suppresses HCC via C-Myc and Bcl-2 Regulation

*c-Myc* is a key oncogene involved in over half of all malignancies; it promotes growth, represses growth-inhibitory genes, and inhibits miR-122 transcription. *c-Myc* transcriptionally represses *Hnf-3b*, an inducer of miR-122 (Figure 1) [29]. miR-122 regulates the c-Myc transcription factors E2F1 and TFDP, thus promoting tumorigenesis via *c-Myc*-inducible lncRNA inactivating p53 and downregulating p53 expression in HCC [30]. Teng et al. reported that *Bcl-2*, an antiapoptotic gene, exerts pro-fibrotic effects in the liver of *miR-122* knockout mice, a prerequisite for HCC development [31]. *Bcl-w*, another antiapoptotic gene belonging to the class of Bcl-2-like survival factors, is upregulated in HCC and serves as a direct target of miR-122 (Figure 1) [32]. Thus, together with the downregulation of p53, the promotion of the antiapoptotic effects of *Bcl-2* and *Bcl-w* facilitates tumor formation in HCC.

### 5.4. miR-122 Targets G9a to Suppress HCC Tumorigenesis

*G9a* is a novel therapeutic target for HCC [33]. It is commonly overexpressed during the development and progression of HCC [34]. The liver-specific deletion of *G9a* and its knockdown using short hairpin RNA reduces tumorigenicity in HCC [35]. Mono- or di-methylation of histone H3 lysine 9 mediated by *G9a* results in the transcriptional repression of various genes, including proapoptotic gene *BclG* (Figure 1) [36]. Although p53 is involved in *BclG* expression, *G9a* is negatively regulated by miR-122 [37].

### 5.5. miR-122 Reduces HCC Metastasis by Targeting TLR4

HCC often involves inflammation and immune evasion. *TLR4* overexpression is a characteristic feature of various cancers, including HCC [38]. Additionally, *TLR4* polymorphism among donors results in the recurrence of HCC in recipients [39]. *TLR4* plays a critical role in carcinogenesis and metastasis in HCC. It confers stemness to tumor cells [40] and enables them to escape from the immune system [38]. *TLR4* is activated via lipopolysaccharide and CD14, initiating signaling via *Myd88*, nuclear factor-κB, mitogen-activated protein kinase, and mechanistic target of rapamycin kinase/Akt, subsequently activating interleukin (IL)-23 and Th17 [38]. Together with IL23 and Th17, proinflammatory cytokines reduce CD^8+^ cell activity in the tumor microenvironment and increase regulatory T-cell (T-reg) immunosuppressive activity (Figure 1) [41]. In addition to enabling immune surveillance evasion, *TLR4* promotes stemness in HCC via the TLR4–SRY-box transcription factor 2-AKT signaling pathway [40]. TLR4 mRNA contains miR-122 seed sequences in the 3′-UTR, and the upregulation of miR-122 significantly reduces *TLR4* expression [42].

## 6. Antimetastatic Effects of miR-122 in HCC

### 6.1. miR-122 Inhibits HCC via Wnt/β-Catenin Suppression

**Figure 2:**
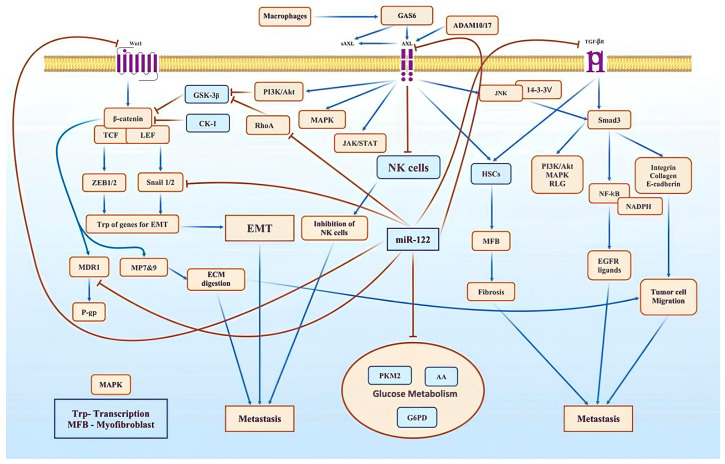
Pathways involved in HCC progression and metastasis.

*Wnt/β-catenin* acts as a growth-promoting signaling pathway in HCC development, inducing the epithelial–mesenchymal transition (EMT) and promoting aggressiveness in tumor cells. It interacts with T-cell and lymphoid enhancer-binding factors (Figure 2) [43]. miR-122 interacts with the 3′-UTR of SNAIL1 and SNAIL2 and decreases their expression in HCC [44]. In addition to downregulating SNAIL1/2, WNT1 is directly targeted by miR-122 [45]. AKT activates the *Wnt/β-catenin* pathway by phosphorylating glycogen synthase kinase-3β. miR-122 inhibits the AKT pathway, whereas *Rho A* promotes glycogen synthase kinase-3β phosphorylation, leading to the increased stability of *β-catenin* (Figure 2) [46].

This figure illustrates the key signaling pathways that contribute to the development and spread of hepatocellular carcinoma (HCC). The *Wnt/β-catenin*, *AXL*, *Rho A*, and *TGF-β* pathways play major roles in these processes.

*Wnt/β-catenin* promotes genes that drive the epithelial–mesenchymal transition (EMT), leading to tumor cell migration and drug resistance.

*AXL* and *Rho A* activate the *β-catenin* pathway, enhancing metastasis and tumor progression.

*TGF-β* stimulates the production of integrins and collagen, which promote cell adhesion and metastasis.

*miR-122* acts as a regulator by inhibiting the *β-catenin*, *AXL*, and *Rho A* pathways to prevent the EMT and metastasis, and directly targeting TGF-β mRNA, reducing its effect, and helping to control tumor progression.

### 6.2. miR-122 Inhibits Fibrosis and HCC via TGF-β Regulation

Hepatic stellate cells (HSCs) in the liver perisinusoidal space protect the liver from injuries, but their persistent activity causes fibrosis and cirrhosis, ultimately leading to HCC [47]. The sustained activation of HSCs is regulated by *TGF-β*, and miR-122 decreases fibrosis by regulating TGF-β [48]. The miR-122-mediated regulation of *TGF-β* is species-dependent; for example, miR-122 targets the 5′-UTR in humans and 3′-UTR in rats (Figure 2) [49]. The improper functioning of *TGF-β* facilitates tumor cell propagation via myofibroblast differentiation, HSC activation, angiogenesis, and abnormal extracellular matrix deposition [50].

### 6.3. miR-122 Inhibits Tumor Invasion via AXL Suppression

The overexpression of *AXL*, a TAM receptor, together with growth arrest-specific 6 (GAS6), an antiapoptosis and pro-cell growth ligand, promotes tumor cell invasion in various malignancies, including HCC [51]. Mechanistically, GAS6/AXL positively regulates Jun N-terminal kinase after interacting with the 14-3-3ζ protein, which stimulates the growth-promoting actions of *TGF-β* via the defective phosphorylation of *SMAD3* (Figure 2) [52]. *AXL* transforms HSCs into myofibroblasts, leading to increased fibrosis in the liver [53]. Furthermore, it facilitates immune evasion by inhibiting natural killer cells, which normally kill tumor cells [54]. Alternately, the ectodomain of *AXL* (sAXL) is released by the action of disintegrin and metalloproteinase proteins (ADAM)-10 and ADAM17 [55]. The intracellular domain of the remaining AXL receptor retains its activity, and sAXL binds with GAS6 [56]. *AXL* facilitates the EMT and tumor invasion by turning on the mechanistic target of rapamycin kinase/Akt, Janus kinase/signal transducer and activator of transcription, nuclear factor-kB, and mitogen-activated protein kinase signaling pathways [57]. miR-122 downregulates *AXL* by directly interacting with its mRNA in the 3′-UTR and inhibiting its metastatic activities (Figure 2) [23].

### 6.4. miR-122 Inhibits HCC Metastasis by Targeting Rho A

*Rho A* is a member of the Rho GTPase (Rho G) family and a key protein in the RAS superfamily that facilitates HCC metastasis [58]. Rho A, along with other factors, facilitates cell propagation via actin polymerization at the leading edge of tumor cells, thus making them motile [59]. Additionally, it induces focal adhesion and stress fibers for attachment to surrounding cells and contractility during migration, respectively [60]. *Rho A* also promotes the EMT by inducing EMT-mediating genes via *AP1* [61]. The Rho A-mediated activation of the extracellular signal-regulated kinase/*p*-38 signaling pathway promotes tumor cell invasion in HCC [62]. Coulouarn et al. revealed *Rho A* as a direct target of miR-122 and demonstrated that miR-122 overexpression reverses the EMT in HCC (Figure 2) [63].

### 6.5. miR-122 Suppresses Tumor Growth by Inhibiting Glycolysis

Cancer cells consume large amounts of glucose for glycolysis and the pentose phosphate pathway, fulfilling their metabolic requirements for cell division. Glucose uptake is facilitated by glucose transporters (GLUTs) [64]. GLUT1, followed by GLUT2 [65], is commonly overexpressed and associated with a poor prognosis in HCC [66]. GLUT1 upregulation is mediated by *c-Myc*, which is indirectly suppressed by miR-122. Glycolytic enzymes aldolase A (AA) [67] and pyruvate kinase M2 (PKM2) [68] are also upregulated in HCC and are associated with the aggressive behavior of tumor cells. Cancer cells require NADPH and ribose for biosynthetic pathways. Glucose 6-phosphate dehydrogenase is overexpressed in various cancers, including HCC [69]. High glucose uptake and its metabolization for NADPH and energy generation increase the survivability and invasiveness of tumor cells. However, AA [67] and glucose 6-phosphate dehydrogenase [69] are directly suppressed by miR-122, thus decreasing glycolysis and arresting cancer cell growth (Figure 2). ADAM10/17 overactivity and *TLR4* upregulation are important factors for EMT in HCC. Lamin B2 and vimentin are often overexpressed and act as miR-122 targets [70].

## 7. The Role of miR-122 in Breast Cancer

### 7.1. The Multifaceted Role of miRNAs in Cancer Progression

Li et al. studied 257 patients with BC and reported the distinct expression of five miRNAs in their plasma (Table 2). They further assessed the diagnostic utility of the miRNAs via receiver operator curve analysis with an area under the curve of 0.683 (95% confidence interval [CI]: 0.597–0.769) in the training set. The area under the curve values of the five miRNAs were 0.966 (CI: 0.940–0.992) and 0.978 (CI: 0.953–1.000) for the internal and external validation sets, respectively. miRNA profiling revealed upregulated miR-122 levels in patients with BC, but the combined panel of five miRNAs enabled better diagnostic prediction. Plasma-derived exosomes only contained upregulated miR-122 [71].

Saleh et al. reported a circulating miR-122 cut-off of >2.2 for differentiation of patients with BC, achieving a sensitivity of 93.33% and specificity of 90% [72]. High circulating levels of miR-122 have better diagnostic predictability than the carcinoembryonic or cancer antigen 15-3. A study showed that high circulating miR-122 levels had 96% sensitivity and 65% specificity for predicting poor clinical outcomes and metastasis in BC [73].

Statistically insignificant differences between the surgical breast tissues of patients with BC and healthy subjects have been reported for miR-122 and ADAM10 levels [74]. Two studies reported low miR-122 expression in BC specimens and cancer cell lines [10]. Similarly, Radojicic et al. reported miR-122 downregulation in 49 specimens of triple-negative BC [75].

### 7.2. Tumor-Suppressing Activity of miR-122 in BC

The overexpression of miR-122 inhibits breast cancer cell growth in vitro and tumor growth in vivo. Moreover, miR-122 levels are downregulated in BC tissues. These findings indicate the tumor-suppressing activity of miR-122 [76]. miR-122 plays a significant role in triple-negative breast cancer (TNBC) by influencing patient survival and gene activity. Research from The Cancer Genome Atlas indicates that patients with low miR-122 levels experience poorer survival outcomes compared to those with higher levels. miR-122 is associated with the regulation of key cancer pathways; high levels correlate with genes involved in cell cycle regulation and DNA repair, which can inhibit cancer progression, while low levels are linked to genes that promote cancer through processes like proliferation and invasion. Furthermore, miR-122 appears to suppress tumor growth by regulating oncogenes, as low levels of miR-122 lead to increased activity of these cancer-driving genes. Overall, miR-122 serves as a potential tumor suppressor in TNBC [77].

### 7.3. miR-122 Suppresses IGF-1R in Breast and Liver Cancers

Abnormally activated *IGF-1R* and its abnormally activated downstream signaling pathways are commonly observed in various cancers, including BC and HCC [78]. miR-122 negatively regulates *IGF-1R* by directly interacting with the 3′-UTR of its mRNA in BC and HCC [23,76].

### 7.4. miR-122 Modulates ADAM10 to Overcome HER2 Resistance

ADAMs are proteases crucial for the cleavage and release of various membrane-anchored proteins, including receptors [79]. The cleavage of the HER2 receptor generates a soluble extracellular domain (ECD) and constitutively active remaining intracellular part. The ECD contains a trastuzumab (TZB)-binding site, which confers ADAM10-mediated TZB resistance [80]. The intracellular part, showing kinase activity, enables ligand-free growth and cell survival. The ECD is released via ADAM10, as indicated by the siRNA-induced inhibition of ADAM10 expression, resulting in decreased shedding of the ECD [81]. ADAMs are overexpressed in various malignancies, including BC and HCC, showing different mechanisms linked to poor patient prognosis [82]. In HCC, ADAM10 and ADAM17 release soluble AXL, which is overexpressed in hepatic fibrosis and HCC [56]. miR-122 regulates ADAM10 by targeting its 3′-UTR, thus decreasing the release of the ECD from the HER2 receptor and overcoming TZB resistance [74].

### 7.5. LncRNA CDKN2B-AS1 Promotes Breast Cancer Growth by Suppressing miR-122

LncRNA CDKN2B-AS1 is associated with various cancers, including BC, exhibiting abnormally high expression [83]. Qin et al. reported that *CDK2NB-AS1* improves cell survival and growth in BC by decreasing the availability of miR-122, thus preventing tumor formation [84].

## 8. miR-122 Is a Pro-Metastatic miRNA in BC

### 8.1. miR-122 Promotes Metastasis via Reverse Warburg Effect

**Figure 3:**
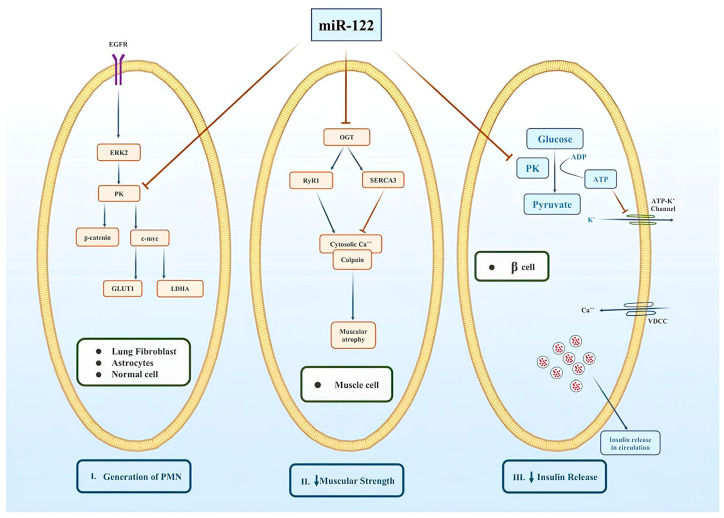
Effects of miR-122 on tumor cells: (**I**) miR-122 targets pyruvate kinase (PK) to decrease glucose uptake by downregulating glucose transporter 1 (GLUT1) expression and promoting pre-metastatic niche (PMN) formation; (**II**) miR-122 targets O-linked N-acetylglucosamine (OGT), increasing cytosolic calcium and promoting muscular atrophy via calpain-mediated degradation of muscular proteins; (**III**) at low PK activity, glycolysis does not generate many ATPs, thereby stopping membrane depolarization in pancreatic β-cells. Lack of membrane depolarization further prevents calcium entry into β-cells, leading to subnormal insulin secretion and hyperglycemia.

The Warburg effect in cancer cells accelerates aerobic glycolysis, decreases mitochondrial oxidative phosphorylation, and promotes cell progression by promoting GLUT1 synthesis via extracellular signal-regulated kinase 2-mediated phosphorylation and PKM2 [85]. BC cells use the reverse Warburg effect to acquire metastatic potential by secreting miR-122-containing exosomes, increasing miR-122 levels, targeting PKM2 mRNA, and decreasing GLUT1 levels and glucose intake (Figure 3) [8]. Cancer cells need high glucose levels for growth, whereas the pre-metastatic niche (PMN) requires less glucose under the influence of exosomal miR-122, thus promoting tumor colonization. Low miR-122 levels promote growth, whereas high circulating miR-122 levels facilitate PMN formation [86].

### 8.2. miR-122 Links Hyperglycemia to Breast Cancer Progression

Women with type 2 diabetes mellitus exhibit a high risk of developing BC. Hyperglycemia and insulin resistance generally lead to poor outcomes in patients with BC [87]. High circulating levels of miR-122 secreted by extracellular vesicles (or exosomes) downregulate PKM in pancreatic β-cells. Decreased PKM activity further reduces ATP generation via glycolysis and leads to the persistent opening of ATP-sensitive K^+^ channels, thereby decreasing insulin release from the pancreas (Figure 3) [88]. The plasma samples of patients with BC exhibit hyperglycemia and decreased c-peptide levels compared with those of normal subjects [89]. Hyperglycemia, combined with decreased glucose uptake by normal cells, helps tumor cells absorb glucose, leading to high tumor cell proliferation and migration in a dose-dependent manner [90]. Therefore, a poor prognosis is positively associated with abnormalities in carbohydrate metabolism in BC.

### 8.3. miR-122 Regulates Calcium Channels in Breast Cancer

The differential expression of two important protein channels, ryanodine receptor 1 (RyR1) and sarcoendoplasmic reticulum calcium ATPase 3 (SERCA3), is vital for the high intracellular calcium concentrations necessary for calpain activation [91]. *RyR1* and *SERCA3* are present in the sarcoplasmic reticulum (SR). The latter aids in Ca^2+^ uptake from the cytosol to the SR, whereas the former releases Ca^2+^ into the cytosol [92]. Therefore, *RyR1* increases cytosolic Ca^2+^ levels [93], whereas *SERCA3* reverses this effect [94]. O-linked N-acetylglucosamine (OGT), an enzyme adding N-acetylglucosamine (GlcNA) to the hydroxyl group of serine or threonine residues of the target protein, mediates a specific post-translational modification regulating the protein turnover of these pumps. Decreased or subnormal O-GlcNAcylation changes the levels of *RyR1* and *SERCA3*. High cytosolic Ca^2+^ levels initiate calpain-mediated proteolysis and myofibril breakdown (Figure 3) [95]. Recently, Yan et al. demonstrated that OGT was a target of miR-122 and reported its decreased activity in the skeletal muscles of BC patients [96].

### 8.4. miR-122 Targets CHMP3 to Regulate Breast Cancer Survival

The MVB pathway degrades growth factor receptors and other proteins in lysosomes. ESCRT-III, including CHMP3, is essential for MVB function. Low CHMP3 expression is associated with a poor prognosis in some cancer types; high CHMP3 expression may indicate improved patient survival. CHMP3 mRNA binds to miR-122 in BC cell lines (MDA-MB-231 and MDA-MB-468) [97,98].

## 9. miR-122 as a Biomarker

Zhang et al. found elevated miR-122 levels in the plasma of patients and animal models with viral, alcohol, and chemical-induced liver disorders. miR-122 offers good sensitivity and specificity for diagnosing liver damage and is readily detectable in blood samples. Thus, miR-122 can be used as a biomarker for liver damage in the diagnosis and monitoring of diseases [99].

Diagnostic studies on miR-122 have reported high sensitivity and specificity when distinguishing between patients with HCV-associated HCC and healthy individuals or those with chronic HCV infection. A meta-analysis showed a pooled sensitivity of 0.87 and a specificity of 0.83 for miR-122 in detecting HCV-related HCC. These findings underscore the potential of miR-122 as a non-invasive and highly accurate biomarker for early HCC diagnosis [100].

Compared with traditional biomarkers like alpha-fetoprotein (AFP), miR-122 has shown superior diagnostic accuracy for detecting HCV-related HCC. While AFP remains widely used in clinical practice, it suffers from limitations such as low sensitivity, especially in patients with early-stage disease. In contrast, miR-122 offers higher sensitivity and specificity, particularly in distinguishing HCV-associated HCC from other liver conditions, such as cirrhosis [101,102].

Despite its promising diagnostic potential, the diagnostic use of miR-122 still faces challenges due to variations in study methodologies, including differences in sample types (serum vs. plasma) and patient demographics. Subgroup analyses suggest that plasma-based tests for miR-122 might provide better diagnostic performance than serum-based tests. Further standardization and validation via larger, multi-center studies are needed to realize miR-122′s potential as a reliable biomarker for HCV-related HCC [100].

## 10. miR-122′s Role in Cancer Treatment

Reduced miR-122 levels in the liver have been associated with the onset of HCC and the enhanced tumorigenicity and metastatic properties of liver cancer cells. Restoring miR-122 expression has been shown to inhibit tumor growth, reduce migration, and induce apoptosis in HCC cells. In vivo studies using animal models further demonstrated that miR-122 replacement therapy can suppress liver tumor growth and improve outcomes, suggesting its potential as a therapeutic agent in liver cancer treatment [103].

miR-122′s therapeutic potential in treating HCC has been demonstrated using various delivery methods, including viral and non-viral systems. One of the most promising approaches involves using an AAV8 vector to deliver miR-122 to liver cells, which has been shown to significantly reduce tumor development in a c-Myc-induced liver cancer model. Additionally, lipid nanoparticle-based delivery systems (LNP-DP1) have been developed to encapsulate miR-122 mimics, providing stability and enhancing uptake by tumor cells. These nanoparticles have proven effective in reducing tumor size in HCC xenograft models. While viral delivery has demonstrated robust and sustained miR-122 expression, non-viral methods are considered safer for clinical applications due to fewer risks of insertional mutagenesis and immune response activation [104].

miR-122′s therapeutic potential extends beyond its tumor-suppressive role. In cases of HCC caused by viral infections, such as hepatitis B virus (HBV) or hepatitis C virus (HCV), miR-122 has demonstrated efficacy in reducing viral replication. For instance, miR-122-targeted therapies have been successful in lowering viral loads in patients with HCV as miR-122 is essential for HCV replication. In HBV-related HCC, restoring miR-122 levels suppresses tumor progression and enhances the effectiveness of standard therapies. These results highlight miR-122 as a promising candidate for combination therapies that target both viral infections and tumor growth in liver cancer [105].

miR-122, a tumor suppressor, modulates tumor proliferation, metastasis, and sensitivity to sorafenib in hepatocellular carcinomas (HCCs). It targets SerpinB3, a molecule that is increased in hepatocellular carcinomas (HCCs) and linked to worse prognosis and resistance to sorafenib. The overexpression of miR-122 enhances susceptibility to sorafenib; yet, resistance remains in SerpinB3-positive cells. The combination of miR-122 restoration treatment and sorafenib has potential in SerpinB3-negative hepatocellular carcinomas (HCCs) [106].

Wang and colleagues’ study showed that miR-122 functions as a tumor suppressor and plays a critical role in inhibiting the formation of new cancers. These criteria indicate that miR-122 could be a new target for therapeutic or diagnostic/prognostic purposes in BC treatment [10,76].

miR-122 has a dual function: it serves as a tumor suppressor in parental breast cancer cells, enhancing radio-sensitivity while acting as an oncomiR in radio-resistant cells. In radio-resistant breast cancer cells, miR-122 is overexpressed, enhancing survival via regulating critical survival pathways. The inhibition of miR-122 mitigates radioresistance. miR-122 targets genes like *ZNF611*, *ZNF304*, and *RIPK1*, which are implicated in the regulation of cell survival, transcription, and resistance pathways such as TNF and Ras-MAPK. Elevated miR-122 expression in patients is associated with improved outcomes after radiation. In radio-resistant cancers, it assumes an oncogenic function, facilitating resistance [107].

## 11. Discussion

The present study investigates the multifaceted roles of miR-122 in the progression and metastasis of hepatocellular carcinoma (HCC) and breast cancer (BC). By consolidating data from numerous peer-reviewed studies, this research contributes to the growing body of literature on microRNAs (miRNAs), specifically miR-122, and their dualistic roles in cancer biology. This discussion interprets the results in light of previous research, explores potential mechanisms of action, and offers insights into future therapeutic directions.

miR-122 has emerged as a critical regulator in both HCC and BC, albeit with opposite effects depending on the cancer type. In HCC, miR-122 functions primarily as a tumor suppressor. It is downregulated in approximately 70% of HCC cases, where it suppresses the expression of genes that promote tumorigenesis and metastasis [10]. The restoration of miR-122 levels has been shown to induce apoptosis and inhibit cell migration, underscoring its potential as a therapeutic target [108]. Conversely, in BC, miR-122 appears to promote metastasis by enhancing glucose metabolism in the pre-metastatic niche [8]. These findings highlight miR-122′s context-dependent roles, which can be exploited for targeted cancer therapies.

A notable finding in this study is the differential expression of miR-122 across cancer types. The downregulation of miR-122 in HCC is consistent with previous studies that have documented its tumor-suppressive role in liver cancer [105]. The mechanisms by which miR-122 exerts this effect include the inhibition of critical pathways such as the Wnt/β-catenin and NF-κB pathways, both of which are involved in the regulation of cell proliferation and survival [10]. In BC, however, miR-122 is upregulated, particularly in metastatic lesions, where it facilitates the metabolic reprogramming of cancer cells, making them more aggressive and capable of surviving in distant organs [109]. miR-122 plays a crucial role in tumor-suppressing tumors like TNBC, influencing cell proliferation and survival mechanisms. It is downregulated in most cases, with lower levels associated with poorer patient outcomes. miR-122 suppresses oncogenes and key signaling pathways, such as *IGF-1R*, which is commonly activated in breast cancer. High miR-122 levels regulate genes involved in cell cycle control and DNA repair, while decreased levels promote cell proliferation, metastasis, and invasion [77].

These results underscore the complexity of miRNA biology, where a single miRNA can play contrasting roles depending on the cellular context. Such findings have significant implications for the development of miRNA-based therapies. For example, therapies aimed at restoring miR-122 expression in HCC could suppress tumor growth, while inhibiting miR-122 in BC might prevent metastasis. This dual approach could provide a more tailored therapeutic strategy, reducing the likelihood of side effects associated with non-specific miRNA modulation [110].

The current findings are consistent with prior research on miR-122 in HCC, where its downregulation is associated with poor prognosis and increased metastatic potential. Previous studies have shown that miR-122 targets genes such as *cyclin G1* and *laminin B2*, both of which are involved in tumor progression [23]. The present study adds to this body of knowledge by providing a comprehensive overview of the molecular mechanisms through which miR-122 exerts its effects, including the regulation of key signaling pathways.

In contrast, the role of miR-122 in BC is more complex and somewhat controversial. While several studies have documented its pro-metastatic role in BC, others have reported conflicting results, particularly concerning its expression levels in primary tumors versus metastatic sites [10]. The current research reconciles these discrepancies by suggesting that miR-122′s role in BC may be context-dependent, with its upregulation occurring primarily in the metastatic niche rather than in the primary tumor. This finding aligns with the concept of the pre-metastatic niche, where cancer cells adapt to distant organs through metabolic reprogramming, a process in which miR-122 plays a pivotal role [8]. Moreover, miR-122, a key gene in breast cancer, has a dual role in radiotherapy. It suppresses tumors in radiosensitive cells, increasing radiation sensitivity, whereas in radio-resistant cells, it promotes survival by modulating pathways like TNF and Ras-MAPK. The overexpression of miR-122 leads to enhanced resistance, but inhibition could reverse this resistance, suggesting that miR-122 modulation could help overcome radiotherapy resistance [107].

The dual roles of miR-122 in HCC and BC open up several avenues for therapeutic intervention. In HCC, miR-122 replacement therapy could be explored as a means to restore its tumor-suppressive functions. Preclinical studies have already demonstrated the feasibility of delivering miR-122 mimics using viral and non-viral vectors, leading to reduced tumor growth and metastasis [111]. Additionally, miR-122 regulates sensitivity to sorafenib in HCC, enhancing cells’ sensitivity by targeting SerpinB3, a gene overexpressed in resistant cases. This restores apoptosis and suppresses metastatic potential, making it a promising treatment for HCC. Combining miR-122 mimics with sorafenib could improve treatment outcomes, especially in patients with SerpinB3-negative tumors [106]. Lipid nanoparticle-based delivery systems have shown promise in enhancing the stability and uptake of miR-122 mimics, suggesting a potential route for clinical translation [112].

In BC, on the other hand, strategies to inhibit miR-122 might be more appropriate. Given its role in promoting metastasis, the development of miR-122 inhibitors could potentially reduce the spread of cancer to distant organs. However, this approach is still in its early stages, and further research is needed to determine the safety and efficacy of miR-122 inhibitors in clinical settings [10].

The diagnostic potential of miR-122 is another area worth exploring. miR-122 has shown high sensitivity and specificity as a biomarker for HCC, particularly in distinguishing HCC from other liver diseases such as cirrhosis [113]. Its use as a non-invasive biomarker in blood-based tests could significantly improve early detection rates, which is crucial for improving patient outcomes [114]. In BC, circulating levels of miR-122 have also been linked to poor prognosis and metastasis, suggesting that it could serve as a biomarker for disease progression [115]. Although miR-122 shows promise as a biomarker, the variability in miRNA detection techniques, such as differences between serum and plasma samples, poses significant challenges to its clinical application [116].

## 12. Limitations

Despite its comprehensiveness, this review has some drawbacks. Publication bias may overstate miR-122′s impact. Long-term clinical data are scarce, limiting our understanding of miR-122 modulation’s clinical effects. Further research is needed to understand miR-122′s context-dependent effects, notably in HCC and breast cancer. Technical difficulties in miRNA quantification and target prediction may have affected the results. Inconsistent miR-122 detection methods may cause study variability. The upstream regulators of miR-122 expression and its interactions with other non-coding RNAs are also unknown. The conclusions are constrained by most studies having focused on specific regions or ethnic groups. Finally, miRNA research quickly evolves; thus, this review may exclude recent discoveries. Regardless of these limitations, this review sheds light on miR-122′s roles in HCC and breast cancer, laying the groundwork for future research and treatment.

## 13. Conclusions and Future Perspectives

This review comprehensively examined the dual roles of miR-122 in the progression and metastasis of hepatocellular carcinoma (HCC) and breast cancer (BC). In HCC, miR-122 predominantly functions as a tumor suppressor, inhibiting key oncogenic pathways and enhancing the sensitivity of cancer cells to treatments like sorafenib. Conversely, in BC, particularly in triple-negative breast cancer (TNBC), miR-122 shows a pro-metastatic role, reprogramming the metabolic environment to favor cancer spread. The therapeutic potential of miR-122 lies in its ability to regulate key cancer-driving processes. Restoring miR-122 levels in HCC has shown promise in reducing tumor growth, enhancing apoptosis, and increasing sensitivity to sorafenib. On the other hand, inhibiting miR-122 in BC could help prevent metastasis by disrupting its ability to prepare pre-metastatic niches and modulate glucose metabolism. miR-122 has shown potential as a biomarker for early detection in both HCC and breast cancer. Preclinical studies using viral vectors and lipid nanoparticles have shown efficacy in this area. Future clinical trials should focus on optimizing delivery methods for safe and effective treatment. Inhibiting miR-122 could prevent tumor spread in metastasis, with the development of specific inhibitors aimed at minimizing off-target effects and improving safety profiles. miR-122 has also been linked to radiotherapy resistance in BC, highlighting the need for further research to modulate this gene to overcome resistance and enhance radiotherapy effectiveness in this cancer. Overall, miR-122 offers promising avenues for therapeutic intervention, but its context-dependent roles require further investigation. Targeting miR-122 may lead to more personalized and effective treatments for HCC and BC, improving patient outcomes.

## Figures and Tables

**Table 1:** Summary of the most critical data on the role of miR-122 in hepatocellular carcinoma (HCC).

Aspect	Details
Function in HCC	miR-122 primarily acts as a tumor suppressor in HCC, regulating key oncogenes and signaling pathways that promote tumor growth, metastasis, and survival.
Downregulation in HCC	miR-122 is downregulated in about 70% of HCC cases, contributing to tumor progression, immune evasion, and treatment resistance.
Key Targets in HCC	miR-122 targets oncogenes including *cyclin G1*, *IGF-1R*, *c-Myc*, *Bcl-w*, and *G9a*, which are involved in cell cycle regulation, survival, and metastasis.
Regulation of apoptosis	By targeting *cyclin G1*, *Bcl-w*, and *IGF-1R, miR-122* promotes apoptosis and cell cycle arrest, inhibiting HCC growth.
Role in sorafenib sensitivity	miR-122 enhances sensitivity to sorafenib by targeting SerpinB3, a factor contributing to sorafenib resistance in HCC. miR-122 restoration therapy shows promise.
Metastasis suppression	miR-122 inhibits metastasis by targeting *TLR4*, *Wnt/β-catenin*, and *Rho A*, which are critical for epithelial–mesenchymal transition (EMT) and metastasis.
Role in metabolism	miR-122 downregulates glycolytic enzymes like GLUT1 and PKM2, reducing glucose uptake by tumor cells and thereby inhibiting cancer cell growth.
Therapeutic potential	miR-122 replacement therapy has shown the potential to reduce tumor growth and enhance chemotherapy efficacy in preclinical models.
Prognostic biomarker	miR-122 is considered a promising biomarker for early diagnosis and prognosis in HCC, with higher specificity than traditional markers like AFP.

**Table 2:** Summary of the most critical data on the role of miR-122 in breast carcinoma.

Aspect	Details
Function in breast cancer	miR-122 plays dual roles: as a tumor suppressor in some contexts and as a pro-metastatic factor, particularly in triple-negative breast cancer (TNBC).
Tumor suppression	miR-122 inhibits tumor growth by downregulating oncogenes and modulating cell cycle control and DNA repair genes. Low miR-122 correlates with poorer outcomes.
Pro-metastatic role	miR-122 enhances metastasis by promoting glucose metabolism reprogramming, facilitating pre-metastatic niche formation, and reducing glucose uptake in normal cells.
Therapeutic resistance	miR-122 contributes to trastuzumab resistance in HER2-positive BC by regulating ADAM10, which affects HER2 receptor shedding.
Role in radioresistance	In radio-resistant BC cells, miR-122 promotes survival by modulating TNF and Ras-MAPK pathways, while its inhibition can reverse radioresistance.
Diagnostic/prognostic biomarker	High circulating levels of miR-122 are linked to poor prognosis and metastasis in BC. It could serve as a non-invasive biomarker for disease progression.
Potential therapeutic target	Targeting miR-122 in metastatic BC may reduce tumor spread while restoring its expression could be beneficial in overcoming drug resistance.
